# The Effect of a Smoking Ban on Hospitalization Rates for Cardiovascular and Respiratory Conditions in Prince Edward Island, Canada

**DOI:** 10.1371/journal.pone.0056102

**Published:** 2013-03-08

**Authors:** Katherine Gaudreau, Carolyn J. Sanford, Connie Cheverie, Carol McClure

**Affiliations:** Epidemiology Unit, Chief Public Health Office, Department of Health & Wellness, Charlottetown, Prince Edward Island; Harvard School of Public Health, United States of America

## Abstract

**Background:**

This is the first study to have examined the effect of smoking bans on hospitalizations in the Atlantic Canadian socio-economic, cultural and climatic context. On June 1, 2003 Prince Edward Island (PEI) enacted a province-wide smoking ban in public places and workplaces. Changes in hospital admission rates for cardiovascular (acute myocardial infarction, angina, and stroke) and respiratory (chronic obstructive pulmonary disease and asthma) conditions were examined before and after the smoking ban.

**Methods:**

Crude annual and monthly admission rates for the above conditions were calculated from April 1, 1995 to December 31, 2008 in all PEI acute care hospitals. Autoregressive Integrated Moving Average time series models were used to test for changes in mean and trend of monthly admission rates for study conditions, control conditions and a control province after the comprehensive smoking ban. Age- and sex-based analyses were completed.

**Results:**

The mean rate of acute myocardial infarctions was reduced by 5.92 cases per 100,000 person-months (P = 0.04) immediately after the smoking ban. The trend of monthly angina admissions in men was reduced by −0.44 cases per 100,000 person-months (P = 0.01) in the 67 months after the smoking ban. All other cardiovascular and respiratory admission changes were non-significant.

**Conclusions:**

A comprehensive smoking ban in PEI reduced the overall mean number of acute myocardial infarction admissions and the trend of angina hospital admissions.

## Introduction

The use of and exposure to tobacco products is a leading cause of preventable death and disability worldwide [1]. Exposure to Second-Hand Smoke (SHS), defined as inhaling tobacco materials from sources other than the smoker's own product, has well known cardiovascular and respiratory health consequences for smoking and non-smoking adults and children [2], [3]. The risk of Acute Myocardial Infarction (AMI) increases in a dose-response relationship with exposure to SHS [4], [5].Exposure to SHS at home has been associated with stroke [6], [7], although a meta-analysis failed to find a significant relationship [3]. SHS exposure has been associated with the onset and severity of pediatric asthma and has been weakly associated with the onset and severity of adult asthma and Chronic Obstructive Pulmonary Disease (COPD) [8], [9].

Smoke-free laws, defined as restrictions on smoking in restaurants, publically accessible spaces and all non-publically accessible workplaces [10], have been shown to reduce exposure to SHS in hospitality workers and the general public [11]–[13], decrease smoking prevalence [14], [15] and improve air quality [16]. In Prince Edward Island (PEI), a smoke-free law was introduced in 2003 with amendments in 2006. The daily smoking rate dropped from 24.5% (95% Confidence Interval (CI) 22.5 to 26.5%) in 2001 to 17.1% (95% CI 15.1 to 19.3%) in 2007–2008 and daily exposure to SHS in public places in the previous month dropped from 13.0% (95% CI 10.9 to 15.4%) in 2003 to 6.4% (95% CI 5.1 to 7.8%) in 2007–2008 according to data supplied by the Canadian Community Health Survey [17].

Smoke-free laws have been repeatedly shown to decrease AMI incidence and admission rates in studies based in the Unites States, Europe and Canada [4], [15], [18]. In a meta-analysis of 11 studies, the incidence rate ratio of AMI cases per 100,000 person-years decreased by 14% (95% CI 11% to 18%) in the 2 months to 3 years after the introduction of a smoking ban, with greater reductions among non-smokers and younger individuals [4].Recent research has shown that the correlation between reduced myocardial infarction rates and smoke-free laws is sensitive to model misspecification, particularly when a linear trend is assumed, and may be subject to publication bias [19]–[21]. Studies examining smoke-free laws and other cardiovascular conditions including stroke and angina, have had inconsistent results [18], [22], [23]. Smoke-free laws have been shown to reduce crude admission rates for asthma in adults and children and COPD in observational studies [18], [22], [23].

Previous work has demonstrated substantial geographic variations in the effects of smoking bans on AMI incidence rates, possibly related to climatic, socio-economic and cultural factors in smoking behaviors [4], [24]. Current Canadian studies on the effect of smoking bans on cardiovascular and respiratory hospitalization rates in Toronto, a major urban area, and on the effect of smoking bans on AMI incidence in Saskatchewan, a dry prairie province do not account for the Atlantic Canadian climatic and socio-cultural context [14], [18]. PEI is Canada's smallest province with a population of approximately 143,000, located in the cool and humid Atlantic Maritime eco-zone with an economic focus on tourism, agriculture and fisheries [25]–[27]. Based on the methods used by Naiman et al., we sought to establish the effect of a comprehensive smoking ban in PEI [18]. Knowledge of changes in hospitalization rates with legislated public health interventions is important for policy makers and health planners working in PEI. Although the proportion of the PEI population in 2005 reporting asthma (8.9% (95% CI 7.6% to 10.1%) was the same as the Canadian average of 8.5% (95% CI 8.3% to 8.6%), the proportion reporting heart disease (6.3% (95% CI 5.4% to 7.2%)) was higher than the Canadian average of 4.9% (95% CI 4.8% to 5.0%) [17]. Minor variations in hospitalization rates can put great stress on a small health care system in terms of cost, hospital bed usage and wait times for services [17]. The primary aim of this ecological observational time series study was to examine changes in cardiovascular and respiratory hospitalization rates before and after the introduction of the *Smoke Free Places Act* in PEI [28]. The secondary aim was to examine for changes by sex or age groupings.

## Methods

### Study design and data sources

The Government of PEI introduced a comprehensive smoke-free law on June 1, 2003. The law banned smoking in all public places and workplaces (with an exception for designated smoking rooms) and prescribed a minimum smoking distance of 4.7 m from doorways and air intake and 2.7 m from doorways on patios [28]. On July 1, 2006, further amendments were introduced in PEI banning smoking on school grounds. Sixteen months after PEI, the Province of New Brunswick (NB) (2006 population = 729,995) enacted a similar smoke-free law on October 1, 2004. New Brunswick had no previous smoke-free law, with the exception of the municipality of Fredericton (2006 population = 85,685) where the smoke-free law came into effect July 1, 2003 [29], [30].

Three cardiovascular conditions (AMI, angina, and stroke) and two respiratory conditions (COPD, and adult and pediatric asthma) were selected for extraction from the PEI Discharge Abstract Database (DAD) from April 1, 1995 to December 31, 2008. The DAD is a nationally validated Canadian database [31] which captures all hospital admissions of PEI residents with Provincial Health Numbers (PHNs). The selected conditions were based on a review of the literature and on common causes for hospital admissions in PEI. In addition, three control conditions (appendicitis, pancreatitis and bowel obstruction) were selected to evaluate changes in general admission rates over time because their etiology had no known relationship with SHS exposure. International Classification of Disease 9^th^ Revision (ICD-9) and ICD-10 codes for the selected conditions were identified because the PEI DAD transitioned between ICD versions in 2001–2002 and both are listed in Table 1. Control admissions to NB acute care hospitals for AMI and appendicitis were extracted using the same ICD codes from the NB DAD from April 1, 2001 to September 30, 2004. NB was chosen as a control province to evaluate underlying trends in admissions because they are a Maritime province that enacted a smoking ban after PEI and they share similar socio-economic, climatic and air pollution characteristics, all known risk factors for cardiovascular and respiratory diseases [25], [26], [32].

**Table 1:** International Classification of Disease (ICD) codes for cardiovascular, respiratory and control conditions.

Condition	ICD-9	ICD-10
Acute Myocardial Infarction	410	I21
Stroke – narrow definition	430, 431, 432, 434, 436	I60, I61, I62, I63.3, I63.4, I63.5, I63.8, I63.9, I64
Stroke – wide definition	430, 431, 432, 433, 434, 436	I60, I61, I62, I63, I64, I65, I66
Angina	413, 411.1	I20
COPD*	491, 492, 494, 496	J41, J42, J43, J44
Asthma	493	J45, J46
Acute appendicitis	540, 541	K35, K37
Bowel obstruction	560	K56
Acute pancreatitis	577.0	K85

* Chronic Obstructive Pulmonary disease.

Admissions due to cardiovascular conditions (AMI, angina, and stroke) were limited to 35 years of age and over to limit cases resulting from cocaine use for AMI and angina and different etiologies for stroke in younger individuals [33], [34]. COPD admissions were restricted to 35 years of age and over to reduce falsely coded patients in the database [35]. Control conditions (pancreatitis, appendicitis and bowel obstruction) were limited to age 35 years and over to make them more comparable to the cardiovascular and respiratory conditions studied. Previous work in tobacco control research has examined adults aged 35 to 74 and has examined the effects of smoke-free laws on adults aged 35 to 64 years and 65 to 74 years as subgroup analyses [7], [15]. The age group analysis allows for the effect of reduced exposure to SHS in the workplace to be identified while reducing analysis problems related to small numbers at individual ages [15]. Asthma admissions were divided into pediatric admissions under 15 years of age and adult admissions over 15 years of age to limit the effect of adolescent smoking on childhood asthma admissions [36]. Mid-year population counts for PEI by age group and sex were obtained from the client registry of PHNs for each year under study [14]. Population counts for NB were obtained by interpolating Statistics Canada Census data from 2001 and 2006 to obtain annual population figures [37].

### Statistical analysis

Cases were divided into time periods based on admission date. The pre-ban period was from April 1, 1995 to May 31, 2003. The post-ban period was from June 1, 2003 to December 31, 2008. Cases occurring in the same individual within 28 days of the first admission were counted as one event. Cases occurring in the same individual more than 28 days apart were counted as separate admissions unless the individual had not left acute care prior to the second event. The analysis also accounted for transfers between PEI and other Maritime hospitals to ensure these were not counted as multiple events.

For each case, age, sex and date of admission were used in the descriptive analysis. Age was grouped into 10-year age groups. Basic demographic characteristics were tabulated and examined for each condition, including sex, age and treatment location. The entire population of PEI with PHNs was considered at risk. Age-based mid-year population counts were used as denominators in calculating admission rates. Monthly and annual admission rates were calculated for the entire population and age and sex subgroups. The crude annual admission rate was calculated for each condition and examined using a basic linear regression model presented in the Supporting Information (Text S1 – Model 1). These results were examined for significant trends (P<0.05) in annual admission rates.

For each condition, the monthly admission rate for the entire study period was modeled using a separate Autoregressive Integrated Moving Average (ARIMA) (p,d,q) monthly time series model. The ARIMA model was fit tested using a Box-Jenkins procedure detailed in the Supporting Information (Text S1) [38], [39]. ARIMA models predict present admission rates using past values and the identified autoregressive, integrated and moving average components of the time series to create a stationary time series and accurately model variables. Autoregressive components reflect the relationship between the current point in the time series and past points to a lag of p and moving average components reflect an average of a window of time q used to smooth the time series. The integrated component is used when a time series has an identified trend and differencing to derive the function that it is generated by and reduce the function's power to 1 is necessary to create a stationary series. We represented the change in mean and change in trend following the 2003 smoking ban separately. Once the ARIMA models were selected for each condition, the 2003 smoking ban was integrated into the model using a dummy variable (0 = pre-ban and 1 = post-ban) to represent the change in mean monthly admission rate immediately following the 2003 smoking ban and a count variable (0 = pre-ban and 1 to 67 = month post-ban) to represent the change in trend of monthly admission rates in the entire period after the 2003 smoking ban [40]. Models were constructed for the 2006 amendments using the same method. The significance level for a change in mean or a change in trend was set to P<0.05 for each disease when assessed without sex and age variables.

Age was regrouped into 35 to 64 years and 65 to 104 years for ARIMA age-specific analysis. ARIMA models for age- and sex-specific datasets were selected using the same process as for the overall dataset. For the four subgroup analyses, statistical adjustment for multiple tests of the significance level was derived from P<α/n where α = 0.05 and n = 4, the number of subgroups using the simple Bonferroni method [41], [42] and thus was set at P<0.0125.

Where significant changes in the study condition monthly admission rates were found, prediction graphs were produced to compare predicted admission rates with the smoking ban using one-step forecasting and without the smoking ban using one-step forecasting up to the time of smoking ban and switching to dynamic forecasting after initiation of the ban. One-step forecasting uses the data on the dependent variable available right up until the time of each prediction while dynamic forecasting uses the data up to a particular time, after which the predicted value is used recursively to make later predictions, resulting a smoother predicted line [43]. Monthly admissions rates from NB were also analyzed using ARIMA models following the same model selection process. All analyses were completed using Stata 10.1/IC© (College Station, TX).

### Ethics Statement

Ethical approval was obtained from the PEI Research Ethics Board prior to completing the analysis.

## Results

Two of the nine PEI hospitals were the treatment location for the majority (79.5%) of admissions. Among all hospital admissions, males were admitted more often for AMI (62.2%), angina (59.7%), COPD (57.3%), and pediatric asthma (64.4%). Female patients comprised the majority of adult asthma admissions (65.2%). Both sexes were equally represented among admissions for stroke, appendicitis, bowel obstruction and pancreatitis. Cardiac admissions (AMI, angina and stroke) peaked in the 65 to 74 year (21.9% to 25.5% of admissions for each condition) and 75 to 84 year age groups (26.8% to 35.8% of admissions for each condition). Adult asthma admissions were progressively more common with increasing age until 85 to 94 years of age whereas COPD admissions peaked at 75 to 84 years of age (35.7%). Pediatric asthma admissions peaked at <5 years of age (62.4%).

**Figure 1:**
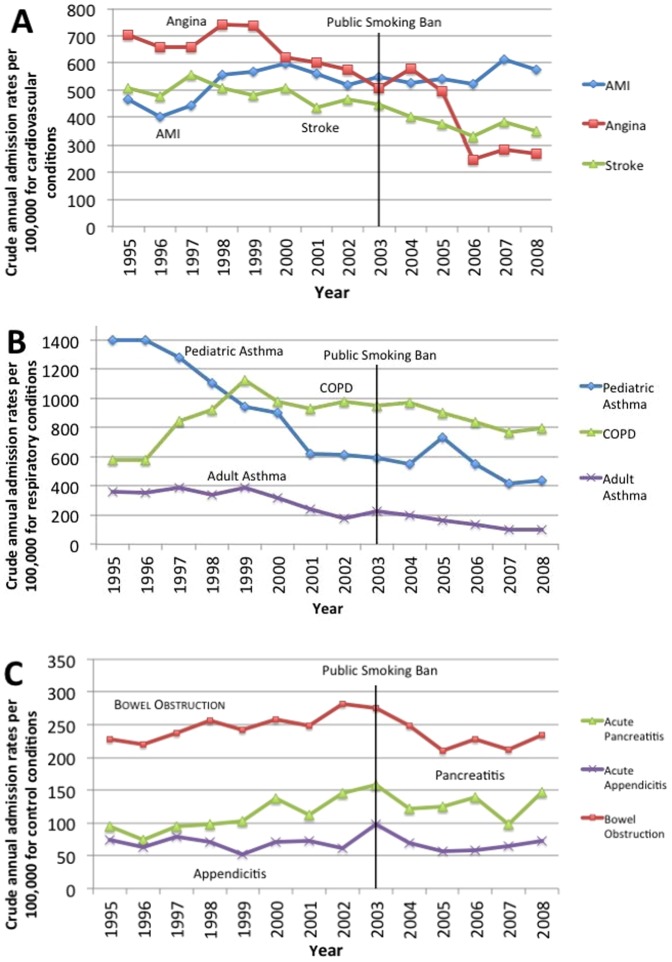
Crude annual admissions to hospital attributable to A) cardiovascular B) respiratory and C) control conditions in PEI, 1995 to 2008. AMI: Acute Myocardial Infarction COPD: Chronic Obstructive Pulmonary Disease.

Crude annual admission rates are presented in Figure 1. Crude annual admissions for angina (P<0.01), stroke (P<0.01), pediatric (P<0.01) and adult asthma (P<0.01) trended downward from 1995 to 2008. Rates for AMI (P = 0.02) and pancreatitis (P = 0.02) trended upward and admissions for COPD (P = 0.43), appendicitis (P = 0.74), and bowel obstruction (P = 0.65) showed no linear trend from 1995 to 2008.

A detailed description of the fitting of each model presented is available in the Supporting Information (Text S1). The cardiovascular and control condition (acute appendicitis, bowel obstruction and acute pancreatitis) graphs did not exhibit any seasonal trends from 1995 to 2008. Both COPD and pediatric and adult asthma showed no seasonal variation, despite the appearance of a possible seasonal trend on the time series graph. On closer examination, COPD, and pediatric and adult asthma had peak admission periods every 7 to 11 months and did not exhibit the strict periodic pattern required to use a seasonal ARIMA model. Large autoregressive components (p) ARIMA models were used instead for COPD and pediatric and adult asthma to model these admission cycles. Both pediatric and adult asthma time series exhibited greater variation in admission rates in the earlier part of the time series and large differences between the mean and median monthly admission rates. A pre-differencing natural logarithm transformation was required to satisfy the ARIMA model condition of equal variance throughout the time series.

The ARIMA model selected for each condition is presented along with the results in Tables 2 and 3. Table 2 describes the change in monthly admission rates following the implementation of a public smoking ban. The mean monthly admission rate for AMI was significantly reduced by 5.92 admissions per 100,000 person-months (P = 0.04) following the 2003 smoking ban. This represents a 13.6% (P = 0.03) decrease in AMI admissions in the month immediately after the smoking ban increasing to a 23.9% (P = 0.03) decrease in December 2008 compared to the pre-ban mean of 43.89 admissions per 100,000 person-months. Angina, stroke, and COPD admissions showed a non-significant decrease in mean monthly admission rates immediately after the 2003 smoking ban and AMI, angina and stroke showed a non-significant decrease in trend after the 2003 smoking ban. Changes in all control diseases were non-significant except there was a small but significant decrease in bowel obstruction rate trend (P = 0.04). All models using the 2006 smoking ban amendment were non-significant (results not shown).

**Table 2:** Change in monthly rates of admission for cardiac, respiratory and control conditions in PEI, per 100,000 population after the smoking ban, 1995 to 2008.

Condition	ARIMA model (p,d,q)	Change in mean monthly admission rate (95% CI)	p-value	Change in trend of monthly admission rate (95% CI)	p-value
*Additive Models*					
AMI*	(0,1,2)	−5.92 (−11.44,−0.39)	0.04#	−0.07 (−0.20, 0.07)	0.32
Angina	(0,1,2)	−3.39 (−19.63, 12.85)	0.68	−0.19 (−0.88, 0.50)	0.59
Stroke	(6,1,0)	−3.04 (−13.14, 7.06)	0.56	−0.05 (−0.75, 0.64)	0.88
COPD †	(10,1,0)	−6.66 (−23.97, 10.64)	0.45	0.22 (−1.15, 1.60)	0.75
Appendicitis	(5,1,0)	6.97 (−3.03, 16.98)	0.17	1.76 (−1.17, 4.69)	0.24
Pancreatitis	(3,1,0)	2.55 (−11.30, 16.42)	0.72	−0.09 (−0.65, 0.47)	0.75
Bowel Obstruction	(0,1,4)	−1.86 (−5.65, 1.93)	0.33	−0.09 (−0.18, −0.005)	0.04#
*Multiplicative Models (ln transformed variables)*					
Pediatric Asthma	(11,1,0)	1.11 (0.63, 1.95)	0.71	1.00 (0.98, 1.02)	0.96
Adult Asthma	(10,1,0)	1.48 (0.90, 2.41)	0.12	0.99 (0.97, 1.01)	0.37

* Acute Myocardial Infarction.

† Chronic Obstructive Pulmonary Disease.

# Significant at P<0.05.

**Table 3:** Change in monthly rates of admission for cardiac and control conditions after the smoking ban, overall and by sex in NB, per 100,000 population, April 1, 2001 to September 30, 2004.

Condition	ARIMA model (p,d,q)	Change in mean monthly admission rate (95% CI)	p-value	Change in trend of monthly admission rate (95% CI)	p-value
AMI*	(0,1,2)	5.84 (−0.80, 12.48)	0.09	−0.61 (−1.35, 0.12)	0.10
Male AMI	(0,1,2)	3.94 (−6.59, 14.47)	0.46	−0.85 (−1.88, 0.18)	0.10
Female AMI	(0,1,2)	6.89 (−2.19, 15.98)	0.14	−0.36 (−1.45, 0.73)	0.52
Appendicitis	(3,1,0)	−0.31 (−2.45, 1.84)	0.78	0.09 (−0.32, 0.50)	0.68
Male Appendicitis	(3,1,0)	−0.10 (−2.01, 1.81)	0.92	0.04 (−0.46, 0.53)	0.89
Female Appendicitis	(3,1,0)	−1.89 (−8.33, 4.55)	0.56	0.21 (−0.34, 0.77)	0.45

* Acute Myocardial Infarction.

Table 4 describes the changes in monthly admission rates by sex. There is a significant change in the trend of angina admission rates in men after the 2003 smoking ban of −0.44 admissions per 100,000 person-months (P = 0.01). This represents a 0.7% (P = 0.94) decrease in monthly angina admissions immediately after the smoking ban, compounding to a 41.8% (P<0.01) decrease in December 2008 compared to the mean pre-ban admission rate of 68.64 admissions per 100,000 person-months. Tables 5 and 6 describe the changes in monthly admission rates by age group. There is a significant (P<0.0125) increase in mean monthly appendicitis cases in females (P = 0.01) following the 2003 smoking ban. Table 3 describes changes in monthly admission rates in NB for AMI and appendicitis. No significant changes in NB admission rates occurred.

**Table 4:** Change in monthly rates of admission for cardiac, respiratory and control conditions after the smoking ban in PEI by sex, per 100,000 population 1995 to 2008.

		Male				Female			
Condition	ARIMA model (p,d,q)	Change in mean monthly admission rate (95% CI)	p	Change in trend of monthly admission rate (95% CI)	p	Change in mean monthly admission rate (95% CI)	p	Change in trend of monthly admission rate (95% CI)	p
*Additive Models*									
AMI*	(0,1,2)	−7.70	0.14	−0.06	0.63	−1.54	0.73	−0.01	0.97
		(−17.87, 2.46)		(−0.32, 0.19)		(−10.27, 7.18)		(−0.35, 0.33)	
Angina	(0,1,2)	0.94	0.88	−0.44	0.01#	−1.99	0.74	−0.19	0.45
		(−11.51, 13.39)		(−0.77, −0.11)		(−13.53, 9.56)		(−0.70, 0.31)	
Stroke	(6,1,0)	1.37	0.87	−0.10	0.86	−7.81	0.29	−0.01	0.98
		(−15.74, 18.49)		(−1.18, 0.98)		(−22.17, 6.56)		(−0.94, 0.92)	
COPD †	(10,1,0)	−11.79	0.26	0.54	0.50	1.67	0.87	−0.03	0.97
		(−32.51, 8.93)		(−1.03, 2.12)		(−18.84, 22.17)		(−1.51, 1.45)	
Appendicitis	(5,1,0)	0.23	0.94	0.11	0.71	3.75	0.01#	−0.16	0.50
		(−5.95, 6.40)		(−0.48, 0.70)		(0.92, 6.58)		(−0.64, 0.31)	
Pancreatitis	(3,1,0)	−1.79	0.63	−0.06	0.86	7.25	0.48	−0.28	0.52
		(−9.04, 5.46)		(−0.71, 0.59)		(−12.79, 27.29)		(−1.14, 0.58)	
Bowel Obs. ‡	(0,1,4)	−2.79	0.27	−0.09	0.09	−1.40	0.61	−0.09	0.21
		(−7.74, 2.17)		(−0.20, 0.01)		(−6.82, 4.03)		(−0.24, 0.05)	
*Multiplicative Models (ln transformed variables)*									
Ped. Asthma#	(11,1,0)	0.97	0.89	1.00	0.86	0.91	0.81	1.01	0.41
		(0.57, 1.62)		(0.97, 1.03)		(0.45, 1.87)		(0.98, 1.05)	
Adult Asthma	(10,1,0)	1.42	0.42	0.99	0.94	1.45	0.17	0.99	0.18
		(0.60, 3.36)		(0.96, 1.03)		(0.85, 2.47)		(0.97, 1.01)	

* Acute Myocardial Infarction.

† Chronic Obstructive Pulmonary Disease.

‡ Bowel Obstruction.

# Pediatric Asthma.

# Significant at the adjusted cut-off, P<0.0125.

**Table 5:** Change in monthly rates of admission for cardiac, respiratory and control conditions after the smoking ban in PEI by age group, per 100,000 population 1995 to 2008.

		35 to 64 years				65 to 104 years			
Condition	ARIMA model (p,d,q)	Change in mean monthly admission rate (95% CI)	p	Change in trend of monthly admission rate (95% CI)	p	Change in mean monthly admission rate (95% CI)	p	Change in trend of monthly admission rate (95% CI)	p
*Additive Models*									
AMI*	(0,1,2)	−3.01	0.16	−0.05	0.32	−9.60	0.52	−0.06	0.88
		(−7.26, 1.23)		(−0.16, 0.05)		(−38.52, 19.32)		(−0.86, 0.74)	
Angina	(8,1,0)	−3.70	0.49	−0.04	0.88	−15.67	0.80	−0.27	0.87
		(−14.16, 6.75)		(−0.60, 0.51)		(−134.9, 103.6)		(−3.37, 2.84)	
Stroke	(6,1,0)	−2.58	0.32	0.02	0.91	−2.83	0.91	−0.36	0.79
		(−7.64, 2.47)		(−0.33, 0.37)		(−51.06, 45.41)		(−2.98, 2.26)	
COPD †	(11,1,0)^1^								
(10,1,0)^2^	−2.16	0.64	−0.10	0.67	−2.25	0.94	1.20	0.58	
		(−11.12, 6.79)		(−0.57, 0.36)		(−56.82, 52.32)		(−3.04, 5.45)	
Appendicitis	(5,1,0)	3.88	0.02	−0.04	0.81	−4.48	0.68	0.98	0.11
		(0.71, 7.05)		(−0.39, 0.31)		(−25.96, 16.99)		(−0.22, 2.19)	
Pancreatitis	(3,1,0)	2.92	0.65	−0.17	0.61	2.62	0.65	0.26	0.68
		(−9.76, 15.60)		(−0.81, 0.47)		(−8.52, 13.76)		(−0.98, 1.50)	
Bowel Obs.‡	(0,1,4)	−0.91	0.56	−0.02	0.52	−6.48	0.18	−0.32	0.02
		(−3.96, 2.13)		(−0.09, 0.04)		(−15.97, 3.00)		(−0.57, −0.06)	

* Acute Myocardial Infarction.

† Chronic Obstructive Pulmonary Disease.

‡ Bowel Obstruction.

1 35 to 64 years.

2 65 to 104 years.

^#^Significant at the adjusted cut-off, P<0.0125.

**Table 6:** Change in monthly rates of admission for asthma after the smoking ban in PEI by age group, per 100,000 population 1995 to 2008.

Age Group	ARIMA model (p,d,q)	Change in mean monthly admission rate (95% CI)	p-value	Change in trend of monthly admission rate (95% CI)	p-value
*Multiplicative Models (ln transformed variables)*					
Pediatric Asthma	(11,1,0)				
0 to 4 years		1.56 (0.05, 44.54)	0.79	1.00 (0.97, 1.04)	0.89
5 to 9 years		0.59 (0.29, 1.20)	0.15	1.02 (0.97, 1.08)	0.37
10 to 14 years		1.20 (0.04, 37.77)	0.92	1.00 (0.90, 1.10)	0.98
Adult Asthma	(10,1,0)				
15 to 34 years		1.64 (0.85, 3.16)	0.14	1.01 (0.97, 1.04)	0.69
35 to 64 years		1.51 (0.83, 3.01)	0.17	0.99 (0.96, 1.03)	0.63
65 to 104 years		1.24 (0.64, 2.41)	0.53	0.99 (0.96, 1.02)	0.44

**Figure 2:**
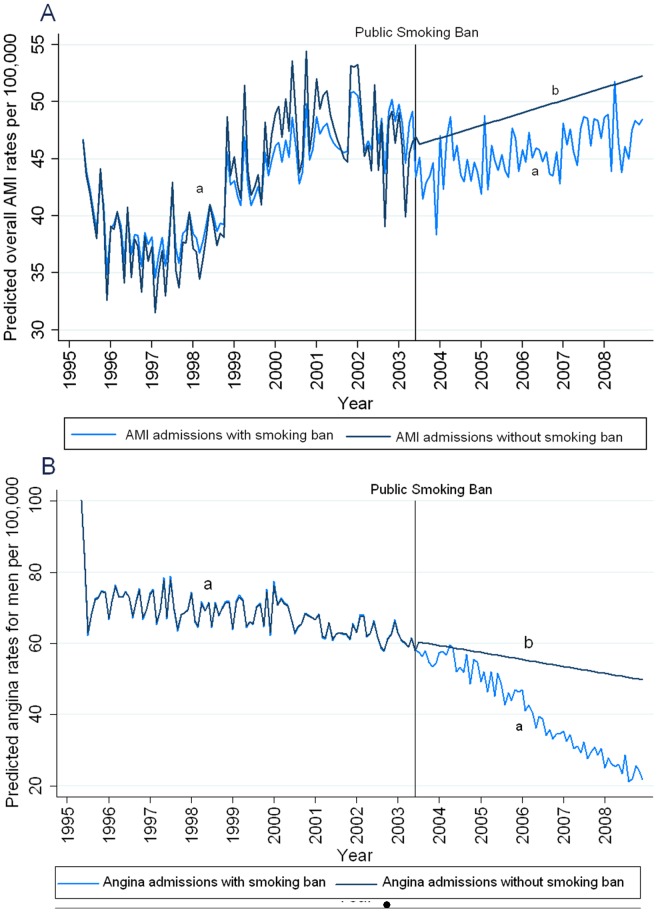
Predicted monthly hospital admission rates in PEI, 1995 to 2008 with and without a smoking ban starting June 1, 2003 for A) overall AMI admissions B) male angina admissions using a) one-step forecasting and b) dynamic forecasting. AMI: Acute Myocardial Infarction.

Figure 2 shows the effect of the changes in admission rates for overall AMI and male angina by predicting the hospital admission rates using models with and without the smoking ban in place. These predictions visually demonstrate the impact of the significant changes in hospital admissions following the smoking ban.

## Discussion

We found a significant decrease in overall mean admissions for AMI and in trend of admissions for angina in men. Although the trends for all cardiovascular diseases decreased non-significantly after the smoking ban, there were no other significant changes found in hospital admission rates for cardiac or respiratory diseases following the smoking ban. Compared with other studies, the difficulty identifying the effects of the smoking ban on hospital admission rates may be due to several reasons. This analysis examined admission rates 8 years prior to and 7 years after the smoking ban, longer than any other study reviewed. Meyers et al. reported that the size of the effect of the smoking ban decreased with the length of time post smoking ban captured by the study [4]. This may account for the many non-significant changes in hospital admission rates in our long-term study. The relatively small size of the population resulted in substantial random variation in monthly admission rates and this may have obscured some of the trend. ARIMA models are excellent tools to deal with the complex correlation structures associated with time series data [38]. The use of ARIMA models allowed changes resulting from the smoking ban to be separated from underlying trends in hospital admissions [40].

All of the cardiovascular and respiratory conditions examined have multiple risk factors such as environmental conditions, physical inactivity, inadequate nutrition, co-morbid conditions, active smoking and second-hand smoke exposure. Due to the nature of the data, no adjustment for these confounding variables was possible and this may have obscured some of the changes in hospital admissions due to the smoking ban. Changes in the distribution of confounders within the population may have changed the risk for admission with cardiovascular and respiratory conditions for the entire population, as well as at the individual level. Further studies examining different levels of these confounders may show additional population and individual level benefits of a comprehensive smoking ban. For example, the increasing levels of obesity and stagnant levels of physical inactivity in PEI from 2001 to 2007–08, well-known risk factors for cardiovascular conditions, may have caused admission rates to be unchanged despite any positive effects of the smoking ban [17]. The predictions for monthly AMI admission rates in PEI with and without a smoking ban (Figure 2A) demonstrate that, even in the setting of increasing hospitalization rates, public health interventions can lower the expected rate of hospitalizations.

The nature of the dataset did not allow for modeling of non-smokers and active smokers separately, despite active smoking being a risk factor for hospital admissions for both respiratory and cardiovascular diseases. It is possible that the observed changes in mean AMI admission rates and trend of angina admissions are the result of more active smokers quitting. Studies have shown that smoking cessation rates increase with a smoking ban and the PEI active smoking rate continues to drop [14], [15], [17]. This drop in active smoking rate is a further benefit of the smoking ban.

Previous studies of cardiovascular and respiratory diseases and smoking bans have recognized and adjusted for the seasonal nature of hospital admissions [15], [36]. No seasonal pattern was apparent in the monthly time series of cardiovascular conditions. The respiratory conditions appeared to have a seasonal pattern on initial examination of the time series graphs, but on further exploration the pattern was irregular with cycles occurring every 7 to11 months. The cause of this irregular variation in admission rates warrants further exploration and could be related to the multiple triggers for exacerbations present in PEI, including agricultural pollens and pesticides, wood burning heat sources, changing air pollution levels, influenza activity and variations in temperature and humidity between seasons.

Ecological studies provide strong evidence for identifying causal associations at the population level and are important tools in evaluating the effect of public health policy [44]. As well, the use of time series models did allow for evaluation of the smoking ban immediately and over time while controlling for time trends present in hospital admission rates before the smoking ban. The use of control conditions and a control province allowed for further comparison of time trends in hospital admissions. There were no decreases in the rates for the controls over the time period except for a small but significant reduction in the trend of admissions for bowel obstruction in all adults. Because of the design of the study, the analysis could not control for other factors such as additional tobacco laws at the federal and provincial levels and public health campaigns that may have affected smoking rates and subsequently hospital admission rates. In the control province (NB), it was not possible to exclude residents of Fredericton, NB who were exposed to a smoke-free law only 1 month after PEI and who represented 11.7% of the control province population. This may have biased the estimate of the effect of the PEI smoking ban towards the null as the changes in hospital admission rates for AMI resulting from the Fredericton smoking ban would have occurred at nearly the same time as in PEI.

This study examined the impact of comprehensive smoking ban within the unique socio-economic, cultural and climatic conditions of Atlantic Canada. This study is one of the first to examine the effect of smoking bans on respiratory disease, stroke, and angina hospital admission rates. The overall AMI results agree with previous research where decreases in overall AMI hospitalizations rates occurred after smoke-free laws were implemented [4], [18]. Our age- and sex- trends in AMI admissions, although not significant, were similar to those found in two Italian studies where men of all ages were more likely to benefit from smoking bans 15,45. Recent studies have suggested that previous findings of significant changes in AMI hospitalizations may be the result of publication bias, model misspecification, including assumptions about linear trends, sampling bias and using too short of a time period after the implementation of a smoking ban [4], [19], [21], [46]. Previous work examining stroke hospitalizations found no significant associations with smoking bans while angina hospitalizations significantly decreased following smoking bans [18], [22], [23]. Men appeared to benefit more from the smoking bans, as they did in two Italian studies showing a greater decrease in AMI admission in men [15], [45]. Similarly, we found a decrease in trend of admissions for angina in men, an associated cardiovascular condition. The current study did not match the findings of previous studies of smoking bans and respiratory conditions that showed large significant decreases in hospitalizations for pediatric and adult asthma and COPD [18], [23], [36]. Shetty et al. have suggested that smoking bans may significantly reduce AMI hospitalizations in areas with limited voluntary private bans and high smoking prevalence but that the effect may be greatly reduced where these conditions do not exist [21]. In addition, we suggest that the effect of the smoking ban in PEI may be reduced by the rural nature of the population leading to a potentially reduced exposure to SHS in public places compared to a more urban setting and by the decreasing active smoking prevalence [17]. The current study was conducted using data from 14 years of hospital admissions, a validated population-wide database, and a robust model identification process, and likely presents a comprehensive picture of the effect of the smoking ban on overall cardiovascular and respiratory hospital admissions in PEI.

## Conclusion

This study provides some of the first evidence of improved health outcomes with the 2003 introduction of smoke-free law in PEI. The smoke-free law introduced on June 1, 2003 was associated with significant reductions in mean monthly AMI admissions and in the trend of angina admissions in men. No other significant changes were found in cardiovascular and respiratory admission rates following the smoking ban. Further research that accounts for individual level confounders such as co-morbidities, diet, physical activity levels and smoking status and environmental factors such as air pollution and climate would provide further evidence for the benefits of smoke-free laws in preventing acute care hospitalizations. The predicted reduced monthly AMI admissions and for angina in men demonstrate that, even when hospitalization rates are increasing, public health interventions can lower the expected rate of hospitalizations.

## Supporting Information

Text S1 **Statistical analysis.**
(DOCX)
