# Supplementary material for: The Effect of a Smoking Ban on Hospitalization Rates for Cardiovascular and Respiratory Conditions in Prince Edward Island, Canada
Source: PLoS One. 2013 Mar 8;8(3):e56102. doi: 10.1371/journal.pone.0056102 (PMC3592861; doi:10.1371/journal.pone.0056102)
Supplement: Text S1 — Statistical analysis. (DOCX) [file pone.0056102.s001.docx]

**Text S1 – Statistical Analysis**

**Linear regression**

*y* = *β_0_* + *β_1_x* + *ε*  (1)

In Model 1, *y* is the annual admission rate and *x* is the year of admission.

**ARIMA (p,d,q) Models**

For conditions with a unit root, including AMI (0,1,2), angina (0,1,2), stroke (6,1,0) and pancreatitis (3,1,0), Model 2 was used:

$\Delta y_{t}=\alpha+\beta_{1}\Delta y_{t-1}+\sum_{p=1}^{p} \rho_{p}y_{t-p}+\sum_{q=1}^{q} \theta_{q}\varepsilon_{t-q}+\delta+\phi+\mu+\varepsilon_{t}$(2)

In the above equation, $\beta_{1}\Delta y_{t-1}$represents the differenced integrated component (*d*); $\sum_{p=1}^{p} \rho_{p}y_{t-p}$ includes all autocorrelation parameters; $\sum_{q=1}^{q} \theta_{q}\varepsilon_{t-q}$ includes the moving-average parameters; $\delta$ is a dummy variable representing pre-ban ($\delta$=0) and post-ban ($\delta$=1) periods;$\phi$ is a count of months after intervention from 1 to 67 (pre-ban months=0) ; $\mu$is a count of months for the entire study period ; and $\epsilon$ is a white-noise disturbance [1]. For appendicitis (0,0,1) and bowel obstruction (0,0,0) without an integrated component, no differenced terms were included (Model 3):

$y_{t}=\alpha+\beta_{1}y_{t-1}+\sum_{q=1}^{q} \theta_{q}\varepsilon_{t-q}+\delta+\phi+\mu+\varepsilon_{t}$(3)

In the final modeling process, these were changed to differenced models using Model 2 for appendicitis (5,1,0) and bowel obstruction (0,1,4) to ensure the change in ban mean and ban trend were being modeled in the same way for all conditions. Finally, for adult (10,1,0) and pediatric (11,1,0) asthma, where a natural logarithm transformation was required, the model of choice is:

$\Delta\ln(y_{t})=\alpha+\beta_{1}\Delta\ln(y_{t-1})+\sum_{p=1}^{p} \rho_{p}y_{t-p}+\delta+\phi+\mu+\varepsilon_{t}$(4)

For COPD, a hinge function was required to account for a change in trend in January of 2000, when COPD admission reached ‘saturation’. The model used for COPD (10,1,0) is:

$\Delta y_{t}=\alpha+\beta_{1}\Delta y_{t-1}+\sum_{p=1}^{p} \rho_{p}y_{t-p}+\sum_{q=1}^{q} \theta_{q}\varepsilon_{t-q}+\delta+\phi+\mu+\lambda+\varepsilon_{t}$(5)

In this equation, $\mu$is a count of months after the ‘hinge’ of January 2000 (pre-hinge months =0).

The mean, range, standard error and median of each time series were examined to determine the shape and spread of the monthly admission rates.

When the variance was more than twice the mean monthly admission rate of the time series, a natural logarithmic transformation of the admission rates was used to stabilize the variance so it was approximately equal throughout the time series. A square root transformation was attempted but did not result in an equal variance throughout the time series.

The transformed (if appropriate) time series was graphed using both raw data and a first order of differencing to detect any nonstationarity that might be corrected with first-differencing. In addition, a linear regression of the monthly admission rate using Model 1 was performed to detect overall trends in monthly admission rates and to guide the selection of the integrated component.

Next, autocorrelation and partial autocorrelation analysis was conducted using the Portmanteau statistic and autocorrelation and partial autocorrelation graphs, in order to assist in determining the optimal number of autocorrelation and moving average parameters. The Augmented Dickey-Fuller (ADF) unit root test was used to detect the presence of a random walk. The ADF test assumes the null hypothesis of a unit root and the alternative of stationarity. The Durbin-Watson statistic and Durbin’s alternative test for autocorrelation were also used to confirm process stationarity and to detect autocorrelation. A variable selection process was used to obtain the highest significant (P<0.05) autocorrelation lag prior to modeling the time series using Akaike’s Information Criterion (AIC) for a series of autoregression to the maximum lag of 15. Selection of the moving average criteria was guided by a visual examination of moving average plots with a smoother of 2, with higher order smoothers applied if the smoother of 2 improved the fit of the time series.

The initial ARIMA model used the information gathered about the initial p, d and q parameters for the model. From there, the series AIC, Portmanteau’s test for white noise and a visual examination of the autocorrelation and/or partial autocorrelation and residual plots were used to guide the final selection of the best fitting model. Values above and below the initial ARIMA model parameters were tested to see if they lowered the series AIC. The final model used was the model with the lowest AIC, a non-significant (P>0.05) Portmanteau’s test for white noise and autocorrelation and/or partial autocorrelation and residual plots within or very close to the 95% reference lines on the plots.

Because of the nature of respiratory conditions and the apparent seasonal variation seen in the monthly time series graphs, monthly (S=12) seasonal multiplicative ARIMA (p,d,q) (P,D,Q,S) models were considered using the same process as above. These can be defined as:

$\Delta\Delta_{12}y_{t}=\alpha+\beta_{1}\Delta\Delta_{12}y_{t-1}+(\sum_{p=1}^{p} \rho_{p}y_{t-p})(\sum_{P=1}^{P} \rho_{P}y_{t-P})+\sum_{q=1}^{q} \theta_{q}\varepsilon_{t-q}+\delta+\phi+\mu+\varepsilon_{t}$ (6)

$\Delta_{12}$is to seasonally difference the monthly time series and $(\sum_{P=1}^{P} \rho_{P}y_{t-P})$is the sum of the seasonal autoregressive components to lag P. Where natural logarithmic transformations were required, this can be represented as:

$\Delta\Delta_{12}\ln(y_{t})=\alpha+\beta_{1}\Delta\Delta_{12}\ln(y_{t-1})+(\sum_{p=1}^{p} \rho_{p}y_{t-p})(\sum_{P=1}^{P} \rho_{P}y_{t-P})+\sum_{q=1}^{q} \theta_{q}\varepsilon_{t-q}+\delta+\phi+\mu+\varepsilon_{t}$ (7)

In completing the age subgroup analyses, small numbers and changes in correlation patterns required the use of different ARIMA (p,d,q) models for both age groups for angina (8,1,0) and for the 35 to 64 year olds for COPD (11,1,0). These models were used to achieve a non-significant white noise test, a lower AIC and reduce significant residuals.

The percent change in admission rates resulting from the ban was calculated using the average monthly pre-ban admission rate and calculating the percent effect of the ban (8) when significant changes in study condition admission rates were found.$\frac{(\delta+(\phi\times months_{post-ban}))}{admit\_rate_{pre-ban}}\times100$(8)

References

1. Stata Corp LP (2007) Stata Release 10 Time Series. College Stations, TX 77845 USA: Stata Press. 448p.
